# Laparoscopic Repair of a Rare Abdominal Wall Deformity and Review of the Literature

**DOI:** 10.7759/cureus.18856

**Published:** 2021-10-18

**Authors:** Anna Sayers, Aggelos Laliotis

**Affiliations:** 1 General Surgery, Royal Alexandra Hospital, Glasgow, GBR; 2 Surgery, Croydon University Hospital, London, GBR

**Keywords:** rare, mesh, hernia, linea arcuate, laparoscopic

## Abstract

Linea arcuate herniae (LAH) are rare and usually asymptomatic but can present with generalized abdominal pain in the absence of bulging and are impalpable. Diagnosis is dependent on cross-sectional imaging, and operative approach to their management is debatable. Here, we report the case of a 56-year-old female with abdominal pain diagnosed with a linea arcuate hernia by computed tomography (CT) scan. She went on to have laparoscopic primary suture closure of the hernial defect with reinforcing underlay mesh. LAH are effectively elucidated with CT. Although there are concerns regarding mesh-related complications, we advocate a laparoscopic approach and repair with prosthetic mesh reinforcement, fixated with sutures. Long-term follow-up of these patients is still required.

## Introduction

Selecting the "best" surgical approach to herniorrhaphy may be contentious enough when encountering common types of hernia. The approach to repairing a rare linea arcuate hernia (LAH), however, poses an even greater dilemma. To the best of our knowledge, there have been only 19 prior cases of symptomatic LAH reported in the literature [1]. There has been an 11:8 female majority with a presenting age ranging between 39 and 79 years [1-3]. The symptoms at presentation are nonspecific abdominal pain and bulging. On the whole, diagnosis is reliant on radiological techniques as it is extremely difficult to identify on clinical assessment alone. When performed in three of the cases, ultrasonography was able to identify a hernia, although it was misdiagnosed as Spigelian hernia in one case. In the remainder of cases, CT was sufficient to confirm LAH; as such, it seems to be a more appropriate first-line test. Interestingly, LAH are a more common occurrence than clinicians may be aware of. A retrospective CT study of 315 unselected patients found a prevalence of 8.57% with a male to female sex ratio of 12.5:1 [2]. This study was randomized but small, and this could be an overestimate.

In no reported instance has MRI been utilized, although it would likely delineate LAH effectively where available. Inferiorly, the rectus abdominis is in direct contact with the transversalis fascia. A LAH is an ascending herniation anterior to transversalis fascia, posterior to the rectus sheath. This poses difficulties for clinical diagnosis as often no hernia can be palpable. Nonspecific abdominal pain has been a common symptom in cases reported hitherto. However, due to the position of the hernia behind the rectus sheath, it seldom causes noticeable bulging, even when frank herniation of peritoneal structures has occurred.

## Case presentation

**Figure 1:**
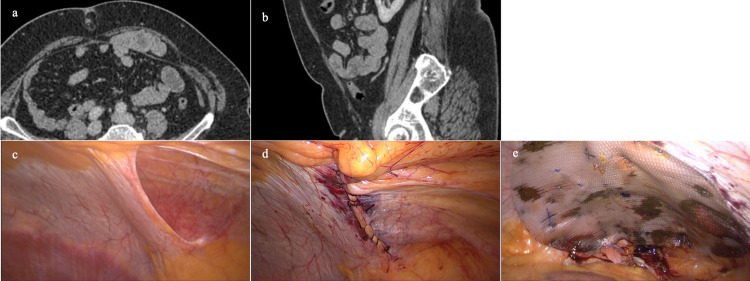
Axial (a) and sagittal (b) computed tomography with intravenous contrast depicting a left-side linea arcuate hernia containing the small bowel. Laparoscopic photographs of the internal defect (c) with primary suture repair (d) and reinforcing prosthetic mesh with hydrogel barrier (e).

A 56-year-old female was seen in the outpatient department with frequent generalized abdominal pain. There were no symptoms of bulging, and on examination, there were no palpable herniae. Laboratory blood tests were unremarkable, and there were normal appearances on oesophagogastroduodenoscopy. Subsequent computed tomography (CT) of the abdomen and pelvis revealed an anterior abdominal wall hernia related to the arcuate line and passing anterior to the transversalis fascia. The appearance was of a linea arcuata hernia (LAH), measuring 95 x 74 mm. It contained a number of loops of the normal small bowel (Figure 1 a-b).

The patient was taken to the theater for elective laparoscopic repair. A 12-mm port and two 5-mm ports were inserted at the right flank at the level of the right midaxillary line. The hernial defect was closed with 2-0 polybutester knotless barbed sutures. Peritoneal fat was dissected to allow reinforcement with a 20 x 15 cm underlay double layer mesh of uncoated medium-weight monofilament polypropylene and absorbable hydrogel barrier secured with an absorbable copolymer strap fixation device (Figure 1 c-e). There has been no recurrence to our knowledge.

## Discussion

Operative management strategies have varied understandably given a prior lack of suitable data and guidance. Open approaches have the distinct disadvantage of a negative exploratory procedure. This is due to the hernia position behind the posterior layer of the rectus sheath failing to produce a bulge. Even when bulging is present, LAH may still be missed intraoperatively unless it is specifically considered and exacerbated by inducing a Valsalva maneuver or even pneumoperitoneum. An open exploratory procedure also risks unnecessarily weakening the rectus sheath and abdominal wall muscle, enlarging the defect and even resulting in an incisional hernia. Seven cases have been successfully repaired laparoscopically, five reinforced with preperitoneal mesh and one with underlay mesh [3,4]. A laparoscopic approach carries the advantage of confirming a diagnosis and allows fixation without the need to perform a laparotomy. Furthermore, the risk of surgical site infection carried by laparoscopic repair is lower than that of open repair [5,6]. This offsets at least some of the additional cost of laparoscopy.

Proponents of an open approach may argue that laparoscopic port site hernia poses an additional unnecessary risk, although the incidence of this complication is very low (up to 0.02%) when fascial closure is maintained for the 10-mm ports in nonobese patients [7], and the rates of an incisional hernia after an open repair are evidently much higher. Recurrence rates after primary ventral hernia and incisional hernia repair are estimated between 15% and 30%, although most studies are lacking in long-term follow-up to give an accurate figure [8]. A large cohort study analyzed 3242 patients undergoing elective incisional hernia repair, and laparoscopic repairs with mesh had the lowest recurrence rate over five years (10.6%) compared with non-mesh repair (17.1%) and open repair with mesh (12.3%) [9]. Moreover, a large study including patients ≥65 years of age undergoing elective open or laparoscopic ventral hernia repair showed that the laparoscopic approach was associated with lower overall morbidity, reduced incidence of surgical site infections, postoperative infections, and reoperation. All other major surgical outcomes were either better in the group treated with laparoscopic surgery or comparable between both treatment groups, except for operative time [10].

To our knowledge, there have been no documented cases of LAH recurrence. The use of mesh in most of the reported cases is probably contributory to this outcome, although longer follow-up periods are required to reach safe conclusions. 

In LAH, we feel that an underlay prosthetic mesh overlapping over 5 cm from the defect edges with an absorbable barrier layer to prevent adhesions has the advantage of providing reinforcement across the suture line and decreases the risk of recurrence. Τhere is also evidence that the risk of recurrence in laparoscopic ventral hernia repairs is decreased with increasing area of mesh overlap [11]. The current Society of American Gastrointestinal and Endoscopic Surgeon and International Endohernia Society guidance advocates laparoscopic mesh fixation using sutures, rather than tacks, for ventral herniae [5,6]. Suture fixation is more cost-effective and has less postoperative pain in the first three months compared with tack use. Rates of recurrence, complication, length of hospital stay, and patient satisfaction, however, are no different. Over time, hydrolysis of the mesh itself, as well as surrounding tissue scarring, causes contraction. Complications of this, such as neuropathic pain and adhesions, are not infrequent. In a large Danish cohort study on patients undergoing elective incisional hernia repair, although recurrence rates were lower when mesh was used, laparoscopic repair resulted in a cumulative incidence of mesh-related complications of 3.7% compared with 5.6% in open repairs [9].

## Conclusions

LAH are rare and usually asymptomatic. They are most common in men, although the symptomatic cases in the literature show a more even sex ratio. They usually present with generalized abdominal pain and are effectively elucidated with CT. Although there are concerns regarding mesh-related complications, we advocate a laparoscopic approach and repair with prosthetic mesh reinforcement, fixated with sutures. Long-term follow-up of these patients is still required. 
